# Organotypic hippocampal culture model reveals differential responses to highly similar Zika virus isolates

**DOI:** 10.1186/s12974-023-02826-6

**Published:** 2023-06-10

**Authors:** Marina da Silva Oliveira, Larissa Marcely Gomes Cassiano, Jeanne Pioline, Ketyllen Reis Andrade de Carvalho, Anna Christina de Matos Salim, Pedro Augusto Alves, Gabriel da Rocha Fernandes, Alexandre de Magalhães Vieira Machado, Roney Santos Coimbra

**Affiliations:** 1 Neurogenômica, Imunopatologia, Instituto René Rachou, Fiocruz, Belo Horizonte, MG Brazil; 2 Departamento de Bioquímica e Imunologia, Universidade Federal de Minas Gerais, Belo Horizonte, MG Brazil; 3 Aix-Marseille University, Marseille, France; 4 Imunologia de Doenças Virais, Instituto René Rachou, Fiocruz, Belo Horizonte, MG Brazil; 5 Plataforma de Sequenciamento NGS (Next Generation Sequencing), Instituto René Rachou, Fiocruz, Belo Horizonte, MG Brazil; 6 Plataforma de Bioinformática, Instituto René Rachou, Fiocruz, Belo Horizonte, MG Brazil

**Keywords:** Zika virus, Organotypic hippocampal culture, Neurodegeneration, Neuroinflammation, Transcriptome, Chromatin remodeling

## Abstract

**Introduction:**

Zika virus (ZIKV) caused an outbreak in Brazil, in 2015, being associated to microcephaly. ZIKV has a strong neurotropism leading to death of infected cells in different brain regions, including the hippocampus, a major site for neurogenesis. The neuronal populations of the brain are affected differently by ZIKV from Asian and African ancestral lineages. However, it remains to be investigated whether subtle variations in the ZIKV genome can impact hippocampus infection dynamics and host response.

**Objective:**

This study evaluated how two Brazilian ZIKV isolates, PE243 and SPH2015, that differ in two specific missense amino acid substitutions, one in the NS1 protein and the other in the NS4A protein, affect the hippocampal phenotype and transcriptome.

**Methods:**

Organotypic hippocampal cultures (OHC) from infant Wistar rats were infected with PE243 or SPH2015 and analyzed in time series using immunofluorescence, confocal microscopy, RNA-Seq and RT-qPCR.

**Results:**

Unique patterns of infection and changes in neuronal density in the OHC were observed for PE243 and SPH2015 between 8 and 48 h post infection (p.i.). Phenotypic analysis of microglia indicated that SPH2015 has a greater capacity for immune evasion. Transcriptome analysis of OHC at 16 h p.i. disclosed 32 and 113 differentially expressed genes (DEGs) in response to infection with PE243 and SPH2015, respectively. Functional enrichment analysis suggested that infection with SPH2015 activates mostly astrocytes rather than microglia. PE243 downregulated biological process of proliferation of brain cells and upregulated those associated with neuron death, while SPH2015 downregulated processes related to neuronal development. Both isolates downregulated cognitive and behavioral development processes. Ten genes were similarly regulated by both isolates. They are putative biomarkers of early hippocampus response to ZIKV infection. At 5, 7, and 10 days p.i., neuronal density of infected OHC remained below controls, and mature neurons of infected OHC showed an increase in the epigenetic mark H3K4me3, which is associated to a transcriptionally active state. This feature is more prominent in response to SPH2015.

**Conclusion:**

Subtle genetic diversity of the ZIKV affects the dynamics of viral dissemination in the hippocampus and host response in the early stages of infection, which may lead to different long-term effects in neuronal population.

**Supplementary Information:**

The online version contains supplementary material available at 10.1186/s12974-023-02826-6.

## Introduction

Zika virus (ZIKV) is an arbovirus of the *Flaviviridae* family, transmitted primarily through the bite of the *Aedes aegypti* mosquito. Infected individuals are usually asymptomatic or oligosymptomatic [1]. In 2015, Brazil faced an epidemic of Zika virus disease cases, initially identified at the Northeast region of the country [2]. In the same period an abnormal increase in the number of cases of congenital microcephaly was observed, a condition that can lead to physical and learning disabilities and intensely affect children development. Later, the exposure of the fetuses to ZIKV during pregnancy and the development of fetal and neonatal abnormalities were associated, being described as ZIKV Congenital Syndrome (ZCS) [3, 4]. Subsequent research revealed that ZIKV infections in both newborns and adults can also lead to cognitive impairments [5–9].

ZIKV is a positive-sense, single-stranded RNA enveloped virus. The genome of approximately 10.8 kb is composed of a long open reading frame and 5' and 3' non-coding regions (UTR) (Reviewed in [10]). Its origins probably date back to Africa between the end of the nineteenth century and the beginning of the twentieth century, spreading in two distinct lineages, the African and the Asian ones [11, 12], the latter being the origin of the isolates circulating in the Americas [13]. In the present study, two isolates of the Asian lineage, with 99.9% similarity at the nucleotide level and 99.97% similarity at the amino acid level, were compared [14]. The first one is PE243 (ZIKV/H. sapiens/Brazil/PE243/2015; GenBank: KX197192), isolated from a patient with classic symptoms associated with Zika, in 2015, in the state of Pernambuco, Brazil [14]. And the second one is SPH2015 (ZikaSPH2015; GenBank: KU321639), isolated from a patient who received blood transfusion from an asymptomatic donor in the state of São Paulo, Brazil [15]. According to Donald et al. [14], PE243 differs from SPH2015 by an A2784G mutation that corresponds to a missense R893G amino acid substitution in the NS1 protein. Two other silent mutations have been reported in the 5’ untranslated region of PE243.

In the context of the central nervous system (CNS), ZIKV can infect neuronal precursor cells as well as immature and mature neurons, inducing cell cycle dysregulation and apoptosis [16–18]. Rich vascularization and proximity to brain lateral ventricles, filled with cerebral spinal fluid, increases the exposure of the circulating virus to the hippocampus compared to other brain structures, making it more susceptible to infection [19, 20]. The hippocampus is located in the temporal lobe and plays a major role in learning and memory [21]. It harbors neural progenitor cells in the subgranular zone (SGZ) of its dentate gyrus (DG) region, which is one of the primary sites of brain neurogenesis in post-natal life [22, 23]. Notably, brain lesions in the fetus, caused by ZIKV infection, are associated with neural stem cells disorganization, loss of progenitor cells and neural circuitry dysmorphia in the DG [24].

Organotypic hippocampal cultures (OHC) can be advantageous over cell culture and organoids to model infection of the central nervous system with ZIKV since it preserves cellular interconnection and cell diversity (Reviewed in [25]), retaining native microglia and the original epigenetic landscape of the different cell types. Also, it allows a remarkable reduction in the number of animals needed, when compared to in vivo models, and enables the investigation of the infection's early effects. OHC from newborn mice have been previously used to demonstrate that ZIKV can replicate in different neuronal populations and kill these cells regardless of their maturation status [26].

This study describes the early impact of the infection with the closely related ZIKV isolates PE243 and SPH2015 on phenotypic features and in the transcriptional signature of the OHC from infant rats and points out some long-term consequences to the hippocampus.

## Materials and methods

### ZIKV

The ZIKV isolates PE243 and SPH/2015 were kindly provided by the Department of Virology and Experimental Therapy (Aggeu Magalhães Research Center/FIOCRUZ) and by the Laboratory of Viral Isolation (Evandro Chagas Institute), respectively. The two isolates were propagated in C6/36 cells and aliquots were stored at − 80 °C in the Laboratory of Immunology of Viral Diseases of the Instituto René Rachou, Fiocruz. The genome regions comprising the M, E, NS1, NS3, NS4A, 2 K, and NS4B genes of the ZIKV isolates in the stocks used in this work were checked by Sanger sequencing (Additional file 1).

### Animals

Neonate Wistar rats (7–10 days old) were obtained from the Institute of Science and Technology in Biomodels (ICTB/FIOCRUZ) and transported to the experimental animal house, at René Rachou Institute/FIOCRUZ, with a lactating female the day before the experiment. Animals were provided with water and food ad libitum, under light/dark cycles of 12–12 h, controlled temperature (20–26 °C) and humidity (40–60%).

### Organotypic hippocampal cultures (OHC)

OHC were produced following the protocol of Stoppini et al. [31] with modifications. After euthanasia, brains were removed and hippocampi were dissected in ice-cold culture medium consisting of MEM Eagle + HEPES (Vitrocell Embriolife, Campinas, Brazil) added with 25% Hank’s balanced salt solution 1x (HBS) (Sigma, Basel, Switzerland). Next, hippocampi were transversally cut at 400 µm using a McIlwain tissue chopper (Mickle Laboratory Engineering Co Ltd, Gomshal, UK) and slices were placed onto culture inserts (PICM0RG50) (Merck Millicell, Darmstadt, Germany) distributed in six-well culture plate containing, in each well, 1 mL of culture medium constituted by MEM Eagle + HEPES (Vitrocell Embriolife) added with 25% HBS 1x (Sigma) and 25% heat-inactivated horse serum (Bio Nutrientes do Brasil Ltda., Barueri, Brazil). OHC were incubated at 37 °C with 5% CO_2_. Every 2–3 days half of the culture medium was changed to renew necessary nutrients. From the seventh day on, heat-inactivated horse serum was suppressed from the medium.

### Infection with ZIKV

After 14 days of culture (stabilization phase), ZIKV infection was performed. OHC were divided into three groups: mock-infected control, infected with ZIKV PE243 and infected with ZIKV SPH2015. For the infection protocol, 1.2 mL of culture medium containing 6 × 10^5^ pfu/mL of the respective virus were used, representing a dilution of approximately 48 × of the viral stocks. Plates remained for two hours in the incubator, with agitation every 20 min. Following, the membranes were washed three times with fresh medium with no virus. The wash procedure ensures that only cell-adsorbed viruses remain in the cultures, preventing a new cycle of infection. Finally, 1 mL of fresh medium was added to the corresponding wells and OHC were incubated until the endpoints of each experiment. Mock-infected controls were also submitted to the same protocol but fresh medium with no virus was used.

### Immunofluorescence

OHC were immune stained as previously described [27]. The density of mature neurons in OHC 8 h, 24 h, 48 h, 5, 7 and 10 days post infection (p.i.) was evaluated using Cy3-conjugated anti-NeuN antibody (1:100, MAB377C3, Merck or 1:100, ABN78C3, Merck). The presence of ZIKV NS1 protein in the OHC was determined 8, 24 and 48 h p.i. with the unconjugated anti-NS1 antibody (1:1000), kindly provided by Dr. Ada Alves from Oswaldo Cruz Institute, Fiocruz. Activated microglia morphology and density in the OHC were estimated 8 h p.i. with the unconjugated anti-Iba1 antibody (1:100, AB178847, ABCAM, Cambridge, UK). Also, the histone H3 trimethylated in the lysine 4 (H3K4me3) was quantified 5, 7 and 10 days p.i. with the unconjugated anti-H3K4me3 antibody (1:500, 05-1339, Merck). Unconjugated antibodies were subsequently labeled with anti-rabbit (1:400, A11034, ThermoFisher, Waltham, MA) or anti-mouse Alexa Fluor 488-conjugated anti-IgG antibody (1:20,000, A10684, ThermoFisher). All OHC were counterstained with 4′,6′-diamino-2-phenyl-indole (DAPI) (1 µg/mL, D1306, ThermoFisher) and slides were mounted with ProLong Diamond Antifade (P36961, ThermoFisher).

### Confocal microscopy

OHC were photographed in a Nikon Eclipse Ti confocal microscope (Nikon, Tokyo, Japan) with a 488/561 wavelength filter, at 10 × or 60 × magnification, searching for representative areas of the tissue, with greater cell densities. Following, images were analyzed using the NIS-Elements Analysis software (Nikon). Initially, channels corresponding to DAPI, NeuN and Iba1 labels were treated with the software tools: *Local Contrast*, *Noise Reduction* and *Gauss-Laplace Sharpen*. Secondly, total tissue area was determined by creating an automatic binary mask, followed using *Interest Region Manager (ROI)* tool, from the channel with DAPI fluorescence. To quantify mature neurons (NeuN +), a binary layer was created, selecting and quantifying elements with a fluorescence threshold > 10 AU, which were later normalized by the tissue area (NeuN + /mm^2^). Similarly, to estimate microglia activation, Iba1 + area values were obtained with the ROI tool and subsequently divided by the total area of the slice (Iba1 + area/mm^2^). The Young et al. [28] Skeleton Analysis approach was utilized to access the branch and ramification lengths of Iba1 + microglial cells. The data were represented as number of endpoints (cell branch tips) divided by the branch lengths and normalized by Iba1 + area. The highest values denoted the least active microglial state (cells with more branched morphology and smaller size). The number of cells with intersection regions between Cy3 and Alexa Fluor 488 channels, corresponding to the number of NeuN + NS1 + cells and the number of NeuN + and TUNEL + cells, were also determined and normalized by the number of NeuN + cells (NS1 + NeuN +/NeuN + and TUNEL + NeuN + /NeuN +, respectively). Additionally, fluorescence intensity (FI) for NS1 (FI NS1/mm^2^) was normalized by tissue area and FI for H3K4me3 was normalized by the number of NeuN + cells (FI H3K4me3 +/NeuN + H3K4me3 + cells).

### RNA samples

Immediately after incubation for 16, 24 or 48 h p.i., depending on the endpoint, the OHC were transferred to RNA-Latter, kept at 4 °C for 24 h and then conserved at − 80 °C until RNA extraction. Total RNA was extracted from pools composed of three OHC from different animals using a combination of Trizol (ThermoFisher) and chloroform (Merck) and purified on a miRNeasy Mini Kit column (217004, Qiagen, Hilden, Germany). Extracted samples were quantified by fluorometry using the Qubit RNA HS Assay Kit (Q32852, Invitrogen, Carlsbad, CA) and the Qubit 2.0 Fluorometer (Invitrogen) and evaluated for their purity with NanoDrop (ThermoFisher). The manufacturers' protocols were followed for every procedure.

### RNA-Seq and bioinformatics

Using the TruSeq Stranded mRNA Kit (Illumina, San Diego, CA), libraries were produced for RNA-Seq using RNA extracted from OHC that had been cultured for 16 h p.i.. The indexed fragments were sequenced using the NextSeq 500 v2 high throughput Kit (75 cycles) (Illumina, San Diego, CA). The software Trimmomatic [29] was used to process raw readings and remove low quality bases (average PHRED score of 20 in sliding window of four bases). Trimmed reads shorter than 50 nucleotides were removed. Clean reads were then aligned to the *Rattus norvegicus* reference genome (release 94) using the software STAR (Spliced Transcripts Alignment to a Reference) [30]. Reads located at a single genomic site were quantified and used in further gene expression analysis [31]. Contrast analysis between infected and control groups were performed using DESeq2 software [32]. Genes with Fold Change > 1.2 and *P* < 0.05 were considered differentially expressed (DEG) and used as input to the functional enrichment analysis performed using the software Ingenuity Pathways Analysis (IPA) (QIAGEN Inc.) with default parameters [33].

### Real-time qPCR validation of differentially expressed genes

cDNA was synthesized using the High-Capacity cDNA Reverse Transcription Kit (ThermoFisher), according to the manufacturer's protocol. qPCR reactions were performed with Fast SYBR™ Green Master Mix (ThermoFisher) using 2 ng/μL of cDNA in a final volume of 10 μL. Rat-specific primers were designed with the aid of Primer-BLAST software (NCBI, Bethesda, MD, https://www.ncbi.nlm.nih.gov/tools/primer-blast/) to detect putative biomarkers identified with the transcriptome analysis (Additional file 2) and *Ppia* was used to normalize gene expression. Thermal cycling and fluorescence detection were performed with the ViiA 7 real-time PCR system (ThermoFisher) according to the manufacturer’s recommendations and relative expression of target genes was calculated using the 2^(− DCt) method [34]. Biomarker candidates were validated at 16 h p.i. Transcriptional expression of *Dbx2*, a negative regulator of neurogenesis, was also assessed in OHC infected with SPH2015 at 24 and 48 h p.i..

### Statistical analysis

Anderson–Darling, Kolmogorov–Smirnov, D’Agostino & Pearson, and Shapiro–Wilk tests were used to evaluate data normality. Unpaired Student’s t test or one-way analysis of variance (ANOVA) with Tukey’s multiple comparison post-test were used to compare groups of parametric data, which were expressed as mean ± standard deviation. Mann–Whitney test or Kruskal–Wallis test with Dunn’s multiple comparison post-test were used to compare groups of nonparametric data, expressed as median and interquartile range. When the comparisons involved the effect of two factors (e.g., time and infection), two-way ANOVA with Tukey’s multiple comparison post-test was used. Differences were considered statistically significant when *P* < 0.05. Statistical analyzes were performed with GraphPad Prism software (version 8.0.2) (GraphPad Software Inc., Irvine, CA).

## Results

### PE243 and SPH2015 differ in two missense mutations

Through Sanger sequencing of the genome regions encompassing the M, E, NS1, NS3, NS4A, 2 K, and NS4B genes, it was confirmed that PE243 differs from SPH2015 due to the A2784G mutation, resulting in a missense substitution of amino acid R893G, as previously reported [14]. Additionally, an extra mutation (G6496A) was identified in the SPH2015 isolate, leading to a missense amino acid substitution (G2165E) in the NS4A protein (Additional file 1).

### PE243 and SPH2015 exhibit different dynamics of infection and proliferation in OHC

**Fig. 1:**
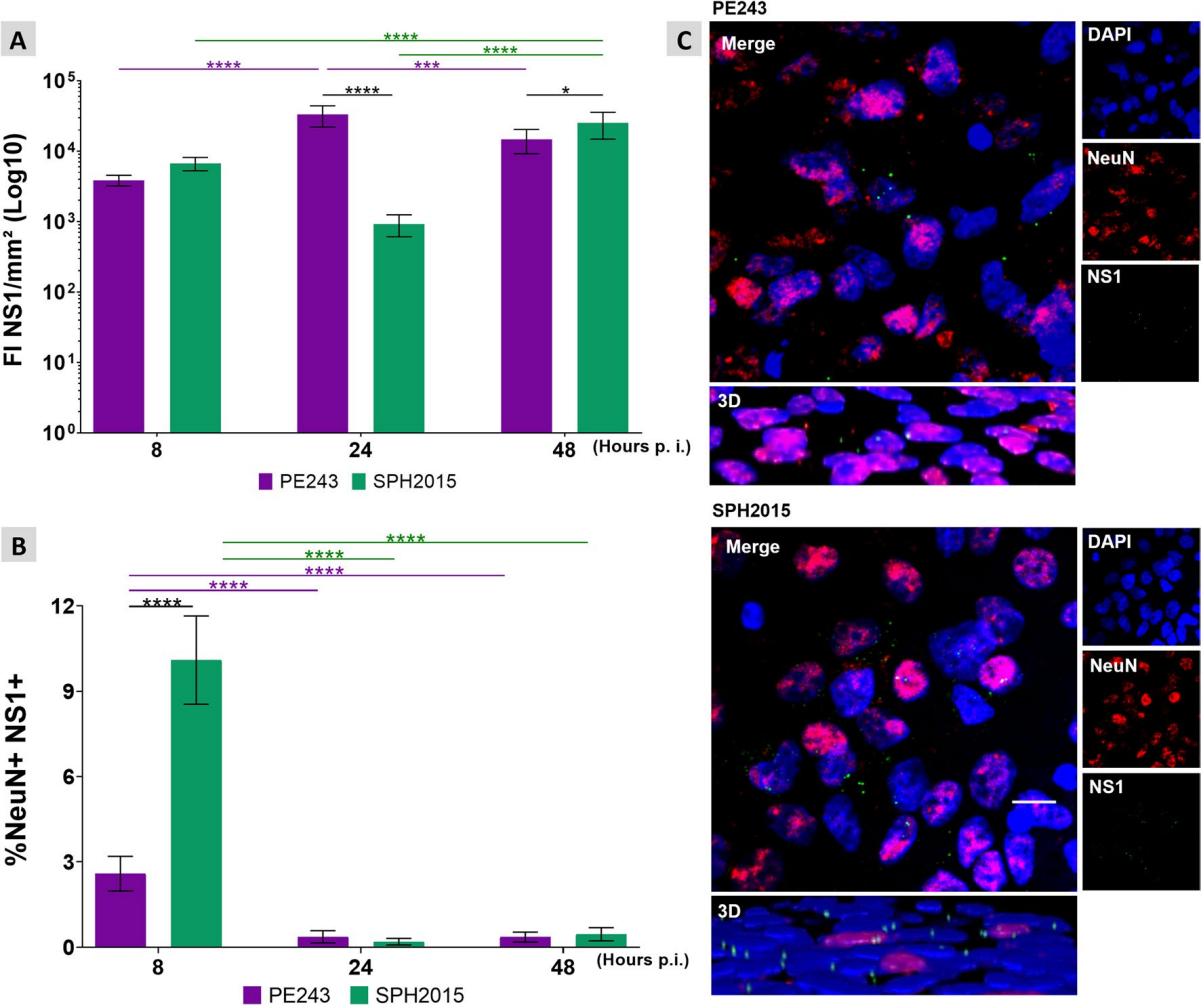
ZIKV infection and proliferation in OHC. **A **NS1 fluorescence Intensity (FI) normalized by tissue area (DAPI +) (FI NS1/mm^2^) was measured for the two isolates 8, 24 and 48 h p.i.. Values outside the range between the lower and upper quartiles of the median of their respective groups (interquartile range) were excluded from the analysis. Two-way ANOVA test was used followed by Tukey’s multiple comparison test. Data were expressed as mean ± standard deviation. *P < 0.05, ***P < 0.01 and ****P < 0.0001. Number of animals = PE243 8 h (7), PE243 24 h (7), PE243 48 h (9), SPH 8 h (11), SPH 24 h (6), SPH 48 h (9). **B **Percentage of mature neurons infected with ZIKV (%NeuN + NS1 +) 8, 24 or 48 h p.i.. Values outside the interquartile range were excluded from the analysis. Two-way ANOVA test was used followed by Tukey’s multiple comparison test. Data were expressed as mean ± standard deviation. ****P < 0.0001. Sample size = PE243 8 h (6), PE243 24 h (7), PE243 48 h (9), SPH 8 h (8), SPH 24 h (6), SPH 48 h (8). **C **Micrographs (60 ×) obtained from OHC 8 h p.i.. Cell nuclei are stained in blue (DAPI), mature neurons in red (NeuN) and ZIKV in green (NS1). In detail, a digital zoom (2,5X) and 3D images show NS1 signals close to the cell nuclei, confirming its intracellular location. Scale bar = 10 µm

The quantification of FI for NS1, a non-structural protein of ZIKV, normalized by the OHC tissue area (FI NS1/mm^2^), revealed that PE243 progressively proliferated in the tissue until 24 h p.i. and then slightly reduced its replication between 24 and 48 h p.i., while SPH2015 reduced its proliferation between 8 and 24 h p.i., to increase it later until 48 h p.i. (Fig. 1A). Thus, different infection and replication dynamics were demonstrated for the two ZIKV isolates tested in the OHC model.

Colocalization of the NS1 and NeuN (%NeuN + NS1 +) proteins showed that both ZIKV isolates infect mature neurons (Fig. 1B). At 8 h p.i., PE243 infected less than 3% of the mature neurons, while SPH2015 was able to infect approximately 10% of these cells. Thus, in the early stages of infection, PE243 has a lower tropism for mature neurons when compared to SPH2015. The percentage of infected neurons substantially decreased after 24 and 48 h p.i. regardless the ZIKV isolate. The NS1 fluorescence signal was detected close to the cell nuclei, confirming its intracellular location (Fig. 1C). RT-qPCR validated the distinct infection and proliferation patterns exhibited by each ZIKV isolate, as observed at the 24 h p.i.. Moreover, they provide conclusive evidence of the presence of detectable viral RNA at six days p.i. (Additional file 3).

### PE243 and SPH2015 caused distinct phenotypic alterations in OHC

**Fig. 2:**
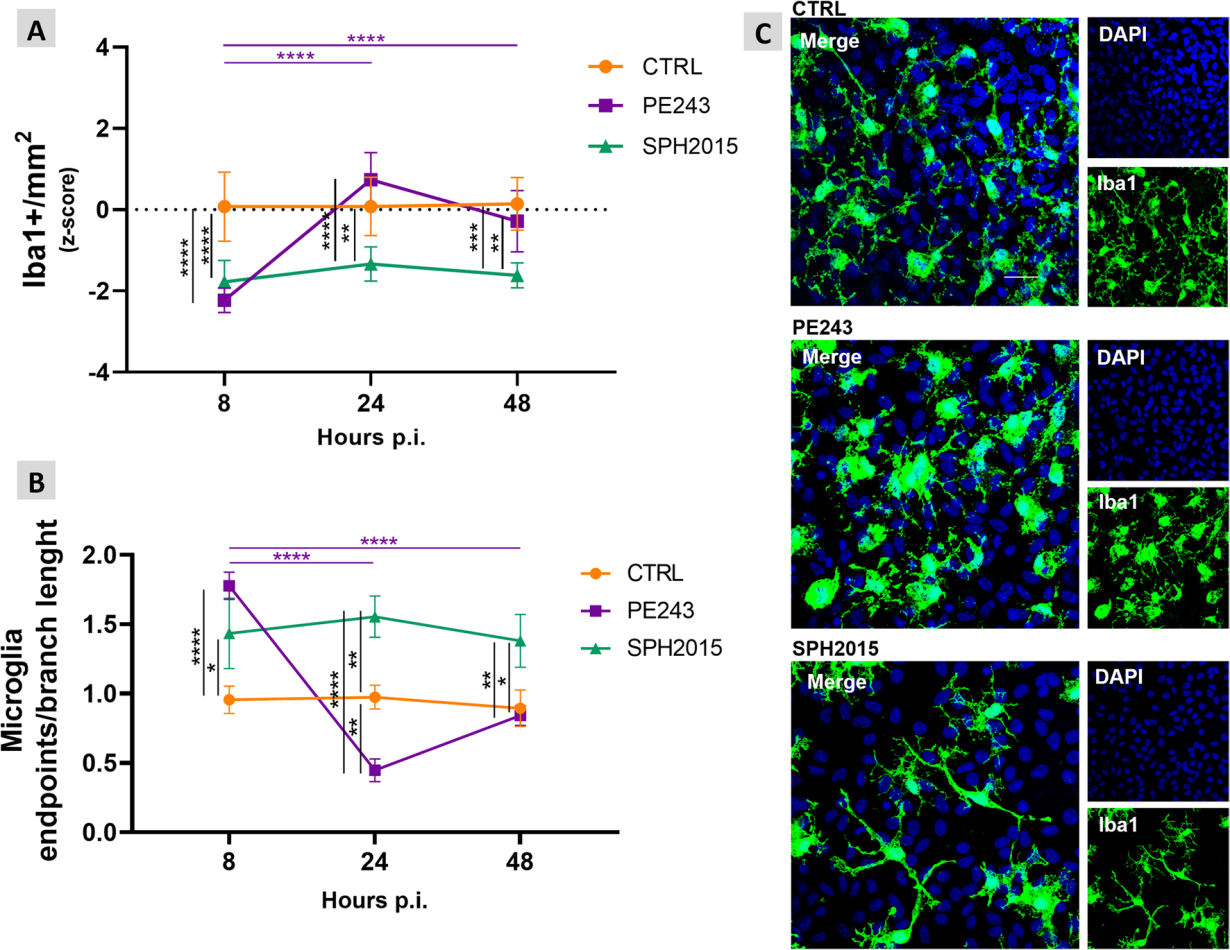
PE243 and SPH2015 have differing effects on microglia in the OHC. **A **The areas corresponding to activated microglia (Iba1 +) were evaluated in mock-infected controls and after infection with ZIKV PE243 or SPH2015 at 8 h, 24 h and 48 h p.i.. Two-way ANOVA test was used followed by Tukey’s multiple comparison test. Statistical significance of *P* < 0.0001 for variations between rows and/or columns (e.g., effect of time, infection, and their interaction). Data were expressed as mean ± standard deviation of Iba1 + /mm^2^ z-scores calculated for the difference between the control and experimental groups in each endpoint. **P < 0.01, ***P < 0.001 and ****P < 0.0001. Number of animals = CTRL (6), PE243 (6), SPH (6) in each endpoint. **B **The means of microglia endpoints/branch length ratio were evaluated to verify microglia activation after infection with ZIKV PE243 or SPH2015 at 8 h, 24 h and 48 h p.i.. Two-way ANOVA test was used followed by Tukey’s multiple comparison test. Statistical significance of *P* < 0.0001 for variations between rows and/or columns (e.g., effect of time, infection, and their interaction). Data were expressed as mean ± standard deviation. *P < 0.05, **P < 0.01 and ****P < 0.0001. Number of animals = CTRL (3), PE243 (3), SPH (3) in each endpoint. **C** Representative micrographs (60 ×) obtained from OHC at 24 h p.i.. Cell nuclei are stained in blue (DAPI) and microglia in green (Iba1). Scale bar = 25 µm

**Fig. 3:**
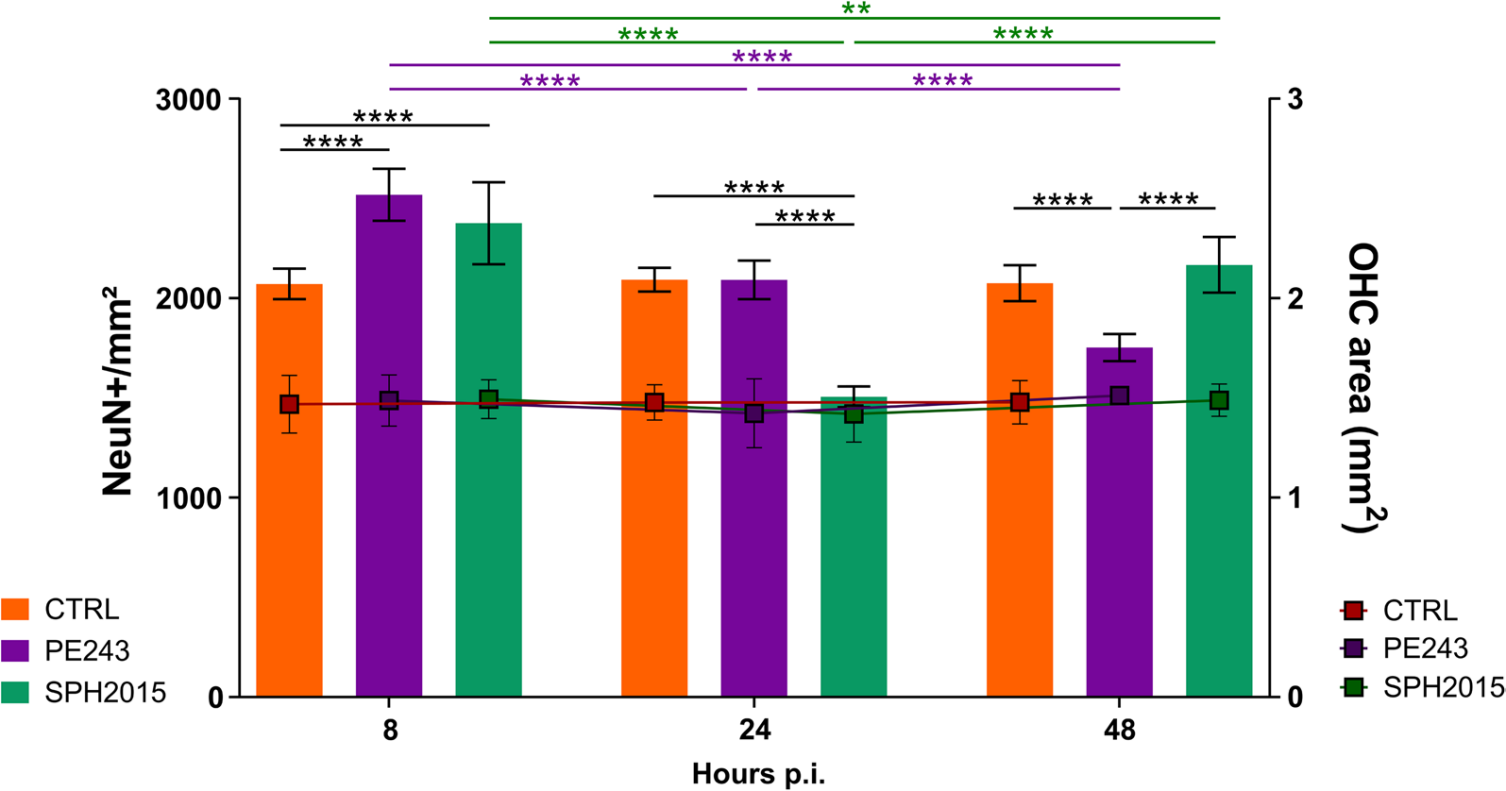
PE243 and SPH2015 have different effects on the neuronal density of OHC within the first 48 h after the infection.** A **The number of mature neurons (NeuN +) per tissue area (DAPI +) was quantified (NeuN + /mm^2^) in the OHC of the control and ZIKV PE243 or SPH2015 infected groups, 8, 24 and 48 h p.i.. Values outside the range between the lower and upper quartiles of the median of their respective groups (interquartile range) were excluded from the analysis. Two-way ANOVA test was used followed by Tukey’s multiple comparison test. Data were expressed as mean ± standard deviation. **P < 0.01; ****P < 0.001. Number of animals = CTRL 8 h (9), CTRL 24 h (8), CTRL 48 h (9), PE243 8 h (9), PE243 24 h (9), PE243 48 h (10), SPH 8 h (12), SPH 24 h (9), SPH 48 h (11)

**Fig. 4:**
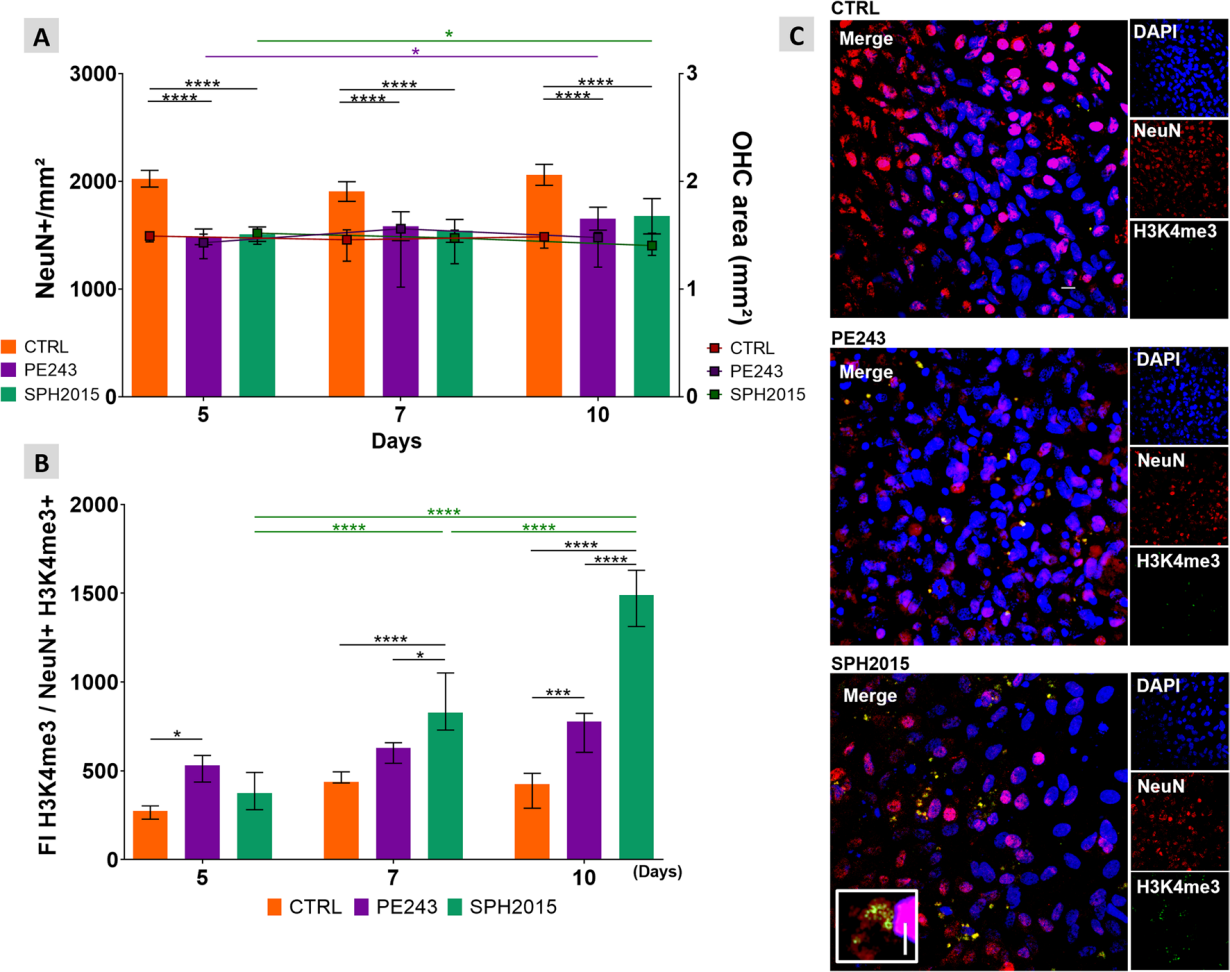
Long-term phenotypic alterations after infection with ZIKV. **A **The number of mature neurons (NeuN +) per tissue area (DAPI +) was quantified (NeuN + /mm^2^) in the OHC of the control and ZIKV PE243 or SPH2015 infected groups 5, 7 and 10 days p.i.. Samples outside the range between the lower and upper quartiles of the median of their respective groups (interquartile range) were excluded from the analysis. Two-way ANOVA test was used followed by Tukey’s multiple comparison test. Data were expressed as mean ± standard deviation. *P < 0.05; ****P < 0.0001. Number of animals = CTRL 5 (13), CTRL 7 (10), CTRL 10 (8), PE243 5 (10), PE243 7 (11), PE243 10 (8), SPH 5 (12), SPH 7 (10), SPH 10 (8). **B **Fluorescence intensity (FI) of the epigenetic marker H3K4me3 was estimated in mature neurons in the OHC of the control and infected with ZIKV SPH2015 and PE243 groups at 5, 7 and 10 days p.i.. These values were normalized by the number of positive neurons for this mark (NeuN + H3K4me3 +) (n ≥ 8). Values outside the range between the lower and upper quartiles of the median of their respective groups (interquartile range) were excluded from the analysis. Two-way ANOVA test was used followed by Tukey’s test of multiple comparisons and data were expressed as mean ± standard deviation. *P < 0.05; ***P < 0.001; ****P < 0.0001. Sample size = CTRL 5 (6), CTRL 7 (6), CTRL 10 (4), PE243 5 (5), PE243 7 (4), PE243 10 (6), SPH 5 (6), SPH 7 (5), SPH 10 (4). **C **Micrographs (60 ×) obtained from OHC 10 days p.i.. Cell nuclei are stained in blue (DAPI), mature neurons in red (NeuN) and H3k4me3 in green. Scale bar = 10 µm. In detail, a digital zoom (2X) of typical H3k4me3 staining (scale bar = 5 µm)

Once it was proved that ZIKV isolates PE243 and SPH2015 infect and proliferate in the OHC model, the impact of the infection on relevant phenotypic aspects of the hippocampal tissue were assessed using immunofluorescence and confocal microscopy. Both isolates led to a reduction in the Iba1 signal in the OHC and to a corresponding decrease in microglia activation, as inferred from their morphology at 8 h p.i., in comparison to the control group. Notably, OHC infected with the SPH2015 isolate maintained this phenotype for up to 48 h p.i.. In contrast, the PE243 isolate induced an increased expression of Iba1 and triggered a morphological transition towards the characteristic phenotype of activated microglial cells, surpassing the control levels of these two parameters at 24 h p.i. and subsequently returning to control levels at 48 h p.i. (Fig. 2). At 8 h p.i., a significant increase in neuronal density was observed in infected OHC when compared to controls, indicating that SPH2015 or PE243 infection induces hippocampal neurogenesis. However, lately, while PE243 induced gradual loss of neurons until 48 h p.i., SPH2015 caused a pronounced loss of these cells between 8 and 24 h p.i., followed by an increase between 24 and 48 h p.i. (Fig. 3). It is reasonable to assume that this is a neurogenic pulse, since the OHC areas remained stable in all groups along the time series, and the expression of the neurogenesis inhibitor *Dbx2* in the cultures infected with SPH2015 was three times lower at 24 p.i. when compared to 48 h p.i., when the OHC were already repopulated with new neurons (Additional file 4). These findings reveal two clearly distinct dynamics of neurodegeneration/neurogenesis caused by ZIKV isolates PE243 and SPH2015 in the first 48 h after the infection. Between five and 10 days p.i., OHC infected with PE243 or SPH2015 exhibited similar neuronal densities, though they were approximately 25% lower than the control group (Fig. 4A). Last but not least, the H3K4me3 epigenetic mark associated with euchromatin and the active state of gene expression was quantified in mature neurons to appraise the potential consequences of ZIKV infection on the functionality of these cells 5 to 10 days p.i.. Intriguingly, SPH2015 led to a progressive and more pronounced increase in the H3K4me3 mark when compared to controls and even PE243 (Fig. 4B). Therefore, these two ZIKV isolates, from the same outbreak and sharing 99.9% similarity at the nucleotide level and 99.97% at the amino acid level [14], have different infection dynamics and induce remarkably distinct phenotypic changes in the OHC.

### Infection with PE243 or SPH2015 affects differently the transcriptome of OHC

**Fig. 5:**
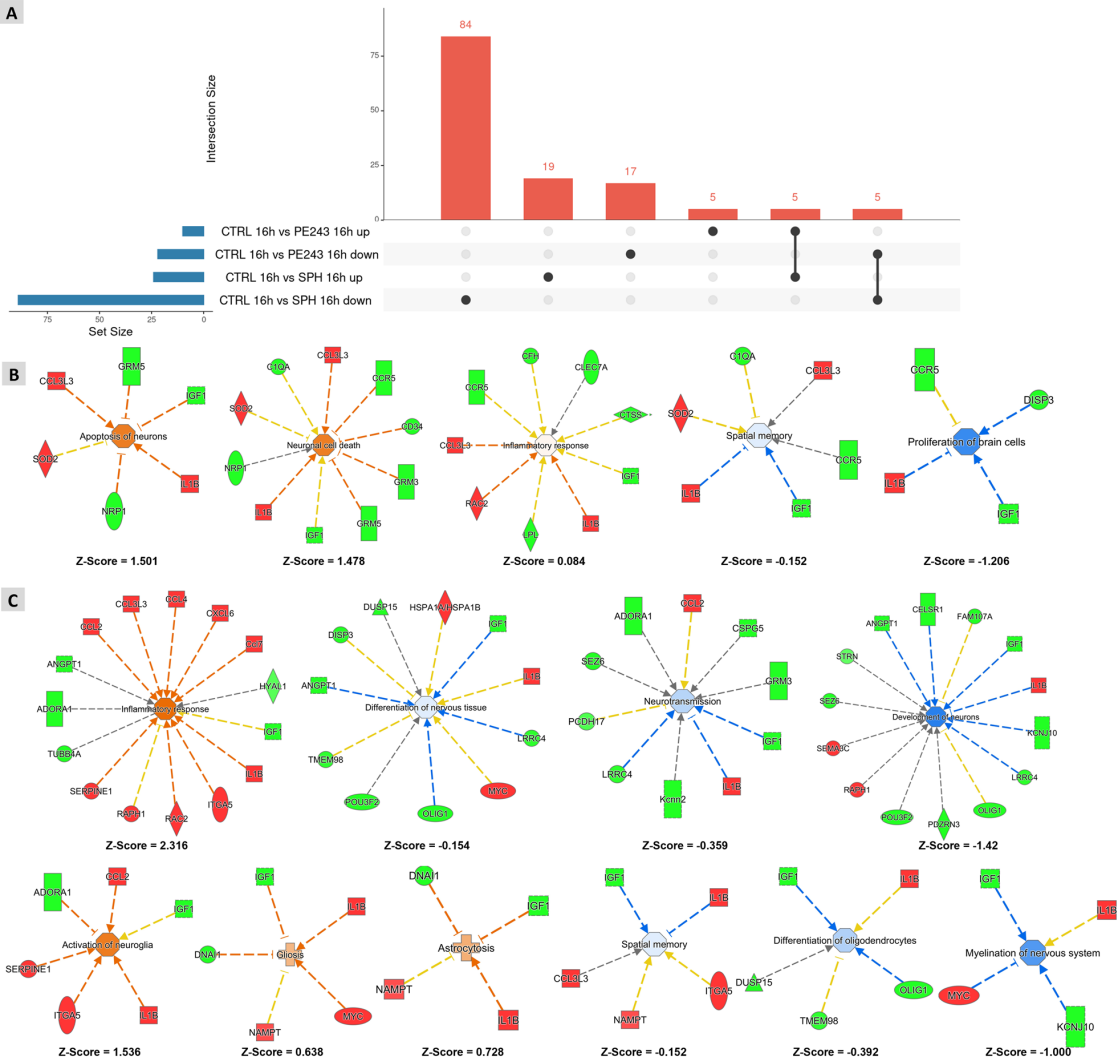
Different effects of PE243 or SPH2015 infection in the OHC transcriptome. **A **Upset plot representing DEGs by OHC infected with PE243 or SPH2015 16 h p.i.. Genes with Fold Change > 1.2 and *P* < 0.05 were considered differentially expressed**. B** and** C **Main biological processes predicted as activated or inhibited by the software IPA based on differentially expressed genes identified after the infection with PE243 (**B**) or SPH2015 (**C**). Filled shapes: in red = activated genes; in green = inhibited genes; in orange = predicted activated process; in blue = predicted inhibited process. Dashed lines: in orange = activation; in blue = inhibition; yellow = inconsistent; arrow shape = induction; T shape = suppression

A transcriptome analysis was performed 16 h after infection to assess the impact of the ZIKV isolates PE243 and SPH2015 on the hippocampal global gene expression at a timepoint in between the neurogenesis (8 h p.i.) and neurodegeneration (24 h p.i.) peaks observed at the immunofluorescence and confocal microscopy. As a result, 32 DEGs (10 upregulated and 22 downregulated) and 113 DEGs (24 upregulated and 89 downregulated) were found in the OHC infected with PE243 and SPH2015, respectively (Fig. 5A and Additional files 5 and 6). Significant differences between the biological processes impacted by infection with each isolate were revealed by functional enrichment analysis of DEGs. These findings essentially account for the phenotypic differences observed.

Both isolates affected the expression of genes that regulate developmental processes in CNS cells (Fig. 5B and C). PE243 upregulated the processes *apoptosis of neurons* and *neuronal cell death*. Biological processes related to cognitive and behavioral development were predicted as downregulated, namely *spatial memory*, by both ZIKV isolates, and *neurotransmission* and *myelination of nervous system*, in response to SPH2015. In addition, infection with SPH2015 downregulated the biological processes *differentiation of oligodendrocytes*, *development of neurons* and *differentiation of nervous tissue*, while PE243 downregulated *proliferation of brain cells*. These results strongly support the phenotypic data that revealed a more prominent reduction on neuronal density between 8 and 24 h in OHC infected with SPH2015 p.i. when compared to those infected with PE243.

Furthermore, depending to the ZIKV isolate, inflammatory DEGs in the OHC varied, and in part corroborate the phenotypic differences observed in microglia after infection with PE243 or SPH2015 (Fig. 2). Functional enrichment analysis of OHC infected with PE243 revealed a mild activation of the process *inflammatory response* (Fig. 5B). For SPH2015, it was disclosed the upregulation of the biological processes of *inflammatory response*, *activation of neuroglia*, *astrocytosis* and *gliosis* (Fig. 5C), indicating a majoritarian role for the astrocytes in the hippocampal response triggered by infection with this isolate.

Both ZIKV isolates regulated similarly 10 DEGs associated with processes such as neuronal death, neurogenesis and inflammatory response, namely: *Adgre1* (adhesion G protein-coupled E1 receptor); *Ccl3l3* (C–C motif chemokine ligand 3 like 3); *Dbx2* (developing brain homeobox 2); *Disp3* (dispatched RND transporter family member 3); *Gmr3* (glutamate metabotropic receptor 3); *Igf1* (insulin like growth factor 1); Il1b (interleukin 1 beta); *Rac2* (Rac family small GTPase 2); *Rasgrp3* (RAS guanyl releasing protein 3); and *Siglec5* (sialic acid binding Ig like lectin 5) (Additional files 5 and 6). These results were confirmed by RT-qPCR in an independent set of samples (Additional file 7).

## Discussion

ZIKV can infect neural progenitors [35], oligodendrocytes [36], microglia [37], astrocytes [38] and mature neurons [39]. It has already been shown that ZIKV from different ancestral lineages cause different effects on the neuronal population of the brain [39]. However, this is the first study to compare the effects of infection in the hippocampus with genetically highly similar ZIKV isolates differing only in two specific missense amino acid substitutions, one in the NS1 protein and the other in the NS4A protein. NS1 is an important non-structural protein that plays a role in viral multiplication and remodeling of the endoplasmic reticulum membrane to establish a replication compartment for the virus. (Reviwed in 40). NS1, which is only produced during viral replication, is important in ZIKV immune evasion, suppressing host response by intervening in the interaction between inflammasome and IFN type 1 signaling [41]. The NS4A protein, in cooperation with NS4B, suppress the Akt-mTOR pathway, resulting in cellular dysregulation [42]. Furthermore, NS4A has been implicated in potential contributions to defects in brain development by inhibiting host ANKLE2 functions [43]. Additionally, NS4A demonstrates antagonistic effects on the host MDA5-mediated induction of the alpha/beta interferon antiviral response [44].

PE243 and SPH2015 successfully infected and replicated in OHC cells, as demonstrated by immunostaining of the NS1 viral protein at early times of infection (Fig. 1A and C). The two ZIKV isolates tested in this study infected mature neurons in early stages of infection (Fig. 1B and C). However, at 8 h p.i., when compared to PE243, SPH2015 showed a much higher infection rate in mature neurons, indicating a higher tropism for these cells. Afterwards, the infection rate of mature neurons decreased for both isolates, suggesting a potential shift in viral tropism towards targeting other cell populations. This observation warrants further investigation to unravel the underlying mechanisms and shed light on this intriguing finding. The differences observed in infectivity or replication between the two isolates were not caused by a decrease in the cell population. This was confirmed by normalizing the findings with the total area of the observed hippocampal cells, which remained constant, as shown in Fig. 3.

The OHC model used herein preserves the microglia, a population of phagocytic and mononuclear cells that reside in the CNS and respond to potentially harmful stimuli by altering morphology and their patterns of proliferation and transcription (Reviwed in 45). Microglia morphology analysis and Iba1 protein quantification from 8 to 48 h p.i. (Fig. 2) suggest that SPH2015 may have more effective host immune system escape mechanisms than PE243. It is worth mentioning that neurogenesis is suppressed by pro-inflammatory cytokines released by activated microglial cells [46], which may have an impact on the neuronal density of OHC infected with ZIKV. Indeed, the population of mature neurons was affected in different ways by PE243 and SPH2015. Initially, at 8 h p.i., the density of mature neurons of the OHC infected with either isolate increased significantly when compared to controls. This increase could be explained by a transient induction of premature differentiation of neural progenitor cells into mature neurons causing a rapid increase in the neuronal population. A similar phenomenon has already been reported in a brain organoid model and may exhaust the pool of neural progenitor cells [47]. However, between 8 and 48 h p.i., the OHC displayed unique patterns of change in their neuronal density in response to PE243 or SPH2015. While the reduction in neuronal density of PE243-infected OHC was continuous and progressive, SPH2015 lead to an abrupt decrease in neuronal population followed by tissue repopulation with new mature neurons (Fig. 3). These findings suggest that PE243, during the period studied herein, may also impair progenitor cells in addition to killing adult neurons, in contrast to SPH2015, which causes more noticeable and sharp neuronal loss but probably spares progenitor neural cells to some extent.

Transcriptome analysis indicates a more robust pro-inflammatory response elicited by SPH2015 infection when compared to PE243 (Fig. 5B and C). In fact, several components of the inflammatory response were upregulated in OHC infected with SPH2015. Several of these genes have been previously associated to the inflammatory response to ZIKV infection [37]. Furthermore, infection of OHC with SPH2015 induced DEGs that support the prediction of activation of astrocytosis and gliosis processes. It is important to note that microglia are not the sole contributors to the inflammatory response within the CNS. While microglia play a significant role in initiating and regulating neuroinflammation, other immune cells, such as astrocytes, also participate in this process. These cell types interact and coordinate their responses, collectively shaping the inflammatory milieu in the CNS. Astrocytosis is an increased proliferation and hypertrophy of astrocytes in response to various pathological conditions in the CNS [48]. Gliosis is a histological finding present in the brains of fetuses with microcephaly [49] and represents a nonspecific reaction of the CNS in response to damage or insults, ultimately contributing to the glial scar formation, which limits edema, but also impedes the regeneration of axons in the affected area [50]. These findings support those of the phenotypic analysis, which suggested that, at least in the early stages of infection, PE243 may have more potent immune system escape mechanisms than SPH2015. In fact, ZIKV has a variety of escape tactics, most of which are related to its non-structural proteins. Mosquito and tick-borne flaviviruses have an sfRNA (subgenomic flavivirus RNA) capable of antagonizing RIG-1-mediated type 1 IFN induction [14]. Also, as previously mentioned, the NS1 protein suppresses the host response by negatively modulating type 1 IFN signaling [41].

Functional enrichment analysis of the DEGs at 16 h p.i. predicted the inhibition of biological processes related to neural progenitor cell proliferation and differentiation, neurotransmission, myelination, and spatial memory. In addition, neuronal apoptosis and neuronal cell death were predicted as activated in PE243-infected OHC (Fig. 5B). Several studies have shown that ZIKV has a tropism for neural progenitor cells, causing decreased neuronal proliferation, cell cycle dysregulation and apoptosis, which could impact fetal CNS development, especially when infection occurs during the first trimester of pregnancy [16, 17, 35]. Infection of areas where postnatal neurogenesis occurs in the adult mouse brain, such as the SGZ of the hippocampus, results in apoptosis of neural precursor cells and reduced proliferation [51]. Altogether, these results indicate that ZIKV can drastically affect the balance between generation and death of neurons in the hippocampus. The mechanisms involved vary according to the viral isolate. While SPH2015 appears to cause neuronal density reduction between 8 and 24 h p.i. negatively regulating neurogenesis and neuron maturation, in the same period, PE243 stimulates the processes of apoptosis of neurons and the death of neuronal cells. Thus, it is plausible to extrapolate that SPH2015 may preserve the pool of progenitor cells of OHC at the beginning of infection, enabling the neurogenesis pulse between 24 and 48 h p.i., which is not observed in OHC infected with PE243.

Infection of OHC with PE243 or SPH2015 commonly regulated a panel of DEGs associated with neurogenesis and inflammatory response (Additional files 5, 6, and 7). Decreased expression of *Disp3* and *Dbx2* genes was observed in infected OHC. Increased expression of *Disp3* stimulates proliferation and inhibits differentiation of neural progenitor cells [52]. Similarly, increased *Dbx2* expression has been shown to inhibit primary neurogenesis in *Xenopus* embryos in vitro [53]. Therefore, inhibition of these genes can stimulate neurogenesis. This expression pattern may be a remnant of the host's genetic programming activated early in the infection, which leads to the phenotype observed at 8 h p.i., when infected OHC have higher neuronal density compared to controls. Also, pivotal inflammatory mediators were upregulated. *Il1b* encodes the cytokine IL-1β and may be related to excitotoxicity and cell death [54]. *Ccl3l3*, related to CCL3, is important in the recruitment of inflammatory cells and has negative effects on synaptic transmission, plasticity and memory in the hippocampus [55]. *Adgre1* encodes the F4/80 antigen, expressed by hippocampal microglia [56], and *Rac2* encodes a member of the superfamily of Rac family small GTPase 2 regulating NADPH oxidase activity, which in turn mediates ROS generation in cells such as phagocytic leukocytes and microglia [57, 58]. And, lastly, *Siglec5* is a member of the Siglec family of surface proteins which is involved in the inhibition of the proinflammatory immune response and phagocytosis in microglia [59]. Other genes were downregulated in response to PE243 or SPH2015. *Igf1* encodes the IGF1 protein, which via its main IGF1R receptor activates the PI3K/AKT (Phosphatidylinositol 3-kinase/Protein kinase B) and MAPK (Mitogen activated protein kinase) pathways, mainly the ERK (Extracellular signal-regulated kinase) pathways. The PI3K/AKT pathway is related to the regulation of processes such as cell proliferation, autophagy and apoptosis and is also important in the antiviral response. Virus-induced activation of PI3K/AKT mediates inhibition of apoptosis, which enables greater potential for viral replication. However, this blockage caused by the virus usually triggers the expression of Interferon-Stimulated Genes. The ERK/MAPK pathway is associated to increased cell differentiation and proliferation (Reviewed in: 60). *Rasgrp3*, also downregulated by ZIKV infection, activates the ERK/MAPK pathway through the Ras protein [61]. Finally, *Grm3* encodes the G protein-coupled glutamate receptor 3 (mGluR3), expressed in neurons and glial cells in regions such as the dentate gyrus of the hippocampus [62]. mGLUR3 is present in pre- and postsynaptic terminals and acts on voltage-gated calcium channels, thus participating in the modulation of neurotransmission [63]. Decreased expression of this gene 16 h p.i. may be a consequence of the loss of neurons observed between 8 and 24 h p.i.. This panel of biomarkers comprises components of the genetic programming underlying the increment in neurogenesis, which was detected up to 8 h p.i., neuronal death, which remarkedly occurred between 8 and 24. p.i., and the inflammatory response at the start of the infection.

At later timepoints, specifically 5, 7, and 10 days post-injury, the OHC had steady but noticeably decreased neuronal density in comparison to controls (Fig. 4A). Even though the OHC’s neuronal density remained stable at similar levels between 5 and 10 days p.i., PE243 and SPH2015 infection affected differently the epigenetic marker H3K4me3 (Fig. 4B), associated with euchromatin and the active state of gene expression [64]. This suggests that the functionality of surviving mature neurons is differently affected depending on the characteristics of the ZIKV isolate. Epigenetic modifications modulate the structure of chromatin or its constituent proteins, resulting in an increased or decreased gene expression [65]. Other members of the *Flaviviridae* family, such as the dengue virus, can induce epigenetic changes in host cells as an immune evasion strategy [66]. However, further studies are needed to clarify these impacts and whether this phenomenon occurs by direct or indirect action of the ZIKV.

## Conclusion

The OHC model has proved to be adequate for the study of the initial stages of ZIKV infection in one of the most important areas of neurogenesis in the CNS. The results here presented indicate that the genetic diversity of the ZIKV isolates is a determinant in the viral ability to infect and proliferate in the hippocampus, in cell tropism, in the profile of the inflammatory response, and in the regulation of the dynamics of neuronal death and neurogenesis in this brain region. The inflammatory response at the beginning of the infection has a central role in determining the effects of ZIKV on the density and, potentially, on the functionality of the surviving neuronal population of the hippocampus. Despite the distinct host responses depending on the genetic profile of the virus, conserved mechanisms were identified, represented by the panel of biomarker genes for neurodegeneration and neurogenesis and the inflammatory response at the beginning of infection.

## Supplementary Information

**Additional file 1.** Sanger Sequencing of PE243 and SPH2015 viral stocks used in this study. Portions of the ZIKV genome encompassing the M, E, NS1, NS3, NS4A and NS4B genes were analyzed, and two mutations were observed, A2784G present in the ZIKV PE243 sequences, as previously reported, and G6496A present in the ZIKV SPH2015 stock used in this study. This new mutation leads to a missense amino acid substitutionin the NS4A protein.

**Additional file 2.** RT-qPCR oligonucleotides. Oligonucleotides used for quantification, by RT-qPCR, of the expression of biomarker genes identified in response to infection with ZIKV isolates PE243 or SPH2015 16 h p.i. in the hippocampus.

**Additional file 3.** Relative quantification of ZIKV RNA by RT-qPCR. Viral load of OHC one and six days after PE243and SPH2015infection were compared pairwise with unpaired t test.

**Additional file 4. Dbx2** mRNA levels after 24 and 48 h of SPH2015 infection. *Dbx2* mRNA levels) 24 and 48 h after SPH2015 infection were compared with two-tailed unpaired t test. Number of animals = 3 per group. Data distribution was evaluated using the Anderson–Darling, D’Agostino & Person, Shapiro–Wilk, or Kolmogorov–Smirnov tests. Data were expressed as mean ± standard deviation. ** *P* ≤ 0.01.

**Additional file 5.** DEGs from OHC infected with ZIKV isolate PE243 16 h p.i. Genes expressed in response to the infection with PE243 or SPH2015 are highlighted in bold.

**Additional file 6.** DEGs from OHC infected with ZIKV isolate SPH2015 16 h p.i. Genes expressed in response to the infection with PE243 or SPH2015 are highlighted in bold.

**Additional file 7. **Real-time PCR validation of putative biomarkers identified with RNA-Seq. Gene expression values) at 16 h post infection for CTRL, PE243 and SPH2015 groups were compared with one-way Analysis of Variancetest followed by Tukey's multiple comparisons analysis. PE243 and SPH2015 were also compared separately with the CTRL group using Student's t testto identify possible statistically significant differences due to the statistical power of the multiple comparison method. Data distribution was evaluated using the Anderson–Darling, D’Agostino & Person, Shapiro–Wilk, or Kolmogorov–Smirnov tests. Outliers were identified by the ROUT test and removed from the analysis. Floating bars were expressed minimum and maximum values. Line at mean. Differences with *P* < 0.05 were considered statistically significant. * *P* ≤ 0.05; ** *P* ≤ 0.01; *** *P* ≤ 0.001; **** *P* ≤ 0.0001; Number of animals = CTRL; PE243; SPH2015.

## Data Availability

The dataset supporting the conclusions of this article is available in the SRA repository (http://www.ncbi.nlm.nih.gov/bioproject/922083).

## Abbreviations

CNS: Central nervous system
DEG: Differentially expressed gene
DG: Dentate gyrus
FI: Fluorescence intensity
HBS: Hank’s balanced salt solution
OHC: Organotypic hippocampal cultures
p.i.: Post infection
pfu: Plaque-forming unit
SGZ: Subgranular zone
ZCS: ZIKV Congenital Syndrome
ZIKV: Zika virus
