# Supplementary material for: Organotypic hippocampal culture model reveals differential responses to highly similar Zika virus isolates
Source: J Neuroinflammation. 2023 Jun 10;20:140. doi: 10.1186/s12974-023-02826-6 (PMC10257278; doi:10.1186/s12974-023-02826-6)
Supplement: Supplementary file 1 — Additional file 1. Sanger Sequencing of PE243 and SPH2015 viral stocks used in this study. Portions of the ZIKV genome encompassing the M, E, NS1, NS3, NS4A and NS4B genes were analyzed, and two mutations were observed, A2784G present in the ZIKV PE243 sequences, as previously reported, and G6496A present in the ZIKV SPH2015 stock used in this study. This new mutation leads to a missense amino acid substitutionin the NS4A protein. [file 12974_2023_2826_MOESM1_ESM.docx]

**Additional file 1**

**Sanger Sequencing of PE243 and SPH2015 viral stocks used in this study**

The amplified regions were obtained using primers adapted from Faria *et al*., 2016, and comprise the regions between 900 to 2846, and 6206 to 7241 of the zika virus genome. These two regions comprise M, E, and NS1, and NS3, NS4A, 2K, and NS4B genes, respectively. RT-PCR was performed with the GoTaq Probe 1-Step RT-qPCR System Kit (Promega, Madison, USA) according to the following protocol: 45 °C for 60 minutes; 95 °C for 2 minutes followed by 35 cycles at 95 °C for 15 seconds, 55 °C for 30 seconds, and 60 °C for 2 minutes. After resolving the amplicons in 1 % agarose gel electrophoresis, samples were purified using the Qiagen Gel Extraction Kit (Qiagen, USA), quantified with Qubit 1X dsDNA HS Assay (Invitrogen, ThermoFisher, Waltham, USA) and approximately 10 ng of DNA was sent for sequencing on the Sanger Capillary Sequencing Platform from Instituto René Rachou - Fiocruz/MG, using the ABI 3730 (Applied Biosystems, ThermoFisher). For Sanger data, the DNA of the samples was sent in duplicates of the primers, the contigs were assembled using the sangeranalyzeR package in R with the parameter to trim at a Phred Score of 20. Multiple sequence alignments were made in the Mega 7 software, using Muscle. All positions in the ZIKV genome were referenced in the Zika PE243 sequence (KX197192.1).

Multiple sequence alignments comparing part of the sequences obtained in this study with the Zika PE243 and SPH2015. The sequences obtained by the Sanger method for this study (PE243_IRR_900_2846; SPH_IRR_900_2846; PE243_IRR_6206_7241; SPH_IRR_6206_7241) were aligned with ZIKV PE243 and SPH2015 sequences from Genbank (KX197192.1_ZikaPE243; KU321639.1_ZikaSPH2015) on Mega 7 software, using Muscle. Portions of the ZIKV genome encompassing M, E, NS1, NS3, NS4A and NS4B genes were analyzed, and two mutations were observed, A2784G present in the ZIKV PE243 sequences, as previously reported (Donald *et al*, 2016), and G6496A present in the ZIKV SPH2015 stock used in this study. This new mutation leads to a missense amino acid substitution (G2165E) in the NS4A protein. No additional difference was observed in other parts of the sequences not shown in the figure. All positions in the ZIKV genome were referenced in the Zika PE243 sequence (KX197192.1).


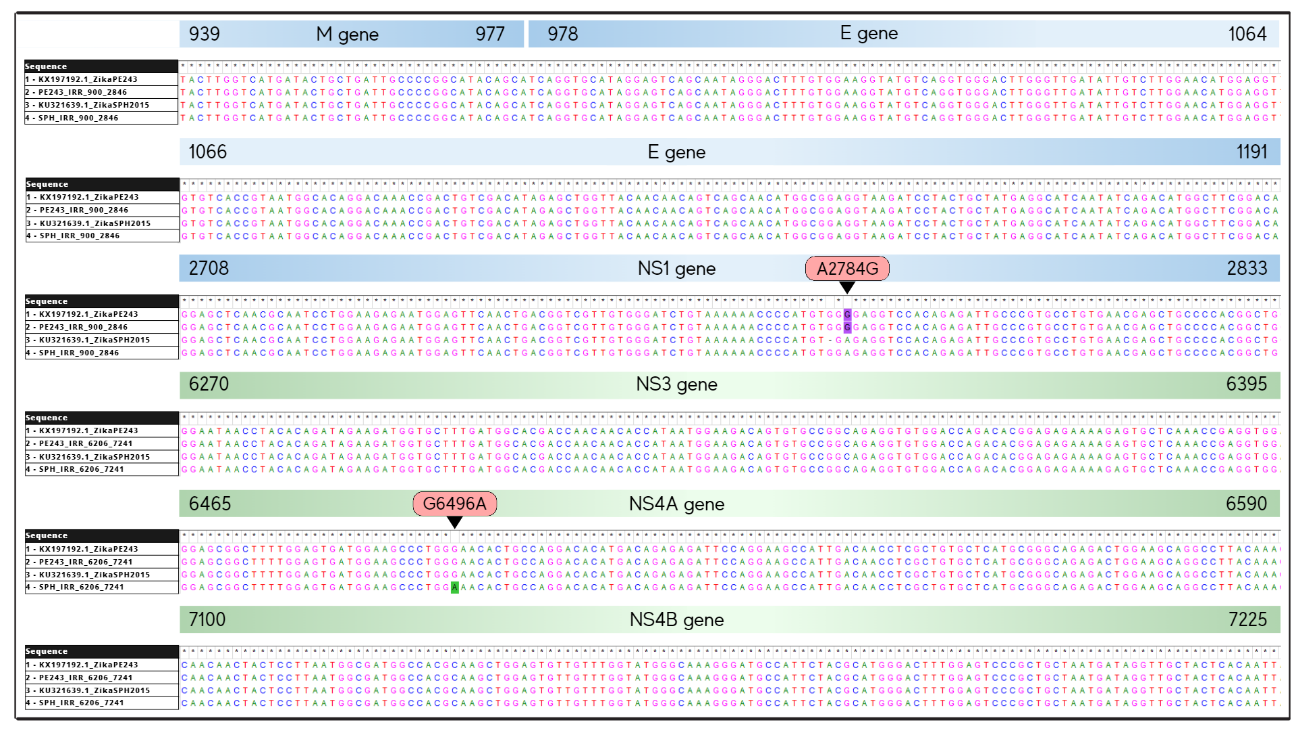


**References**:

Faria, Nuno Rodrigues, et al. Zika virus in the Americas: early epidemiological and genetic findings. *Science* 352.6283 (2016): 345-349.

Donald CL, Brennan B, Cumberworth SL, Rezelj VV, Clark JJ, Cordeiro MT, et al. Full Genome Sequence and sfRNA Interferon Antagonist Activity of Zika Virus from Recife, Brazil. PLoS Negl Trop Dis. 2016;10(10):e0005048
