# Supplementary material for: Organotypic hippocampal culture model reveals differential responses to highly similar Zika virus isolates
Source: J Neuroinflammation. 2023 Jun 10;20:140. doi: 10.1186/s12974-023-02826-6 (PMC10257278; doi:10.1186/s12974-023-02826-6)
Supplement: Supplementary file 2 — Additional file 2. RT-qPCR oligonucleotides. Oligonucleotides used for quantification, by RT-qPCR, of the expression of biomarker genes identified in response to infection with ZIKV isolates PE243 or SPH2015 16 h p.i. in the hippocampus. [file 12974_2023_2826_MOESM2_ESM.docx]

**Additional File 2**

Oligonucleotides used for quantification, by RT-qPCR, of the expression of biomarker genes identified in response to infection with ZIKV isolates PE243 or SPH2015 16h p.i. in the hippocampus

| **Gene Symbol** | **mRNA RefSeq** | **Type** | **Sequence (5'->3')** | **Product lenght** | **Length** | **Start** | **TM (ºC)** | **GC%** | **Exon boundary** |
| --- | --- | --- | --- | --- | --- | --- | --- | --- | --- |
| *Adgre1* | NM_001007557.2 | Primer F | CCCTTGGCAAGCATAATGGC | 107 | 20 | 2618 | 59.89 | 55 | 20 - 21 |
|  |  | Primer R | TATTCATCTCGTACCTGGCGG |  | 21 | 2724 | 59.39 | 52 |  |
| *Ccl3* | NM_013025.2 | Primer F | GCCGGGTGTCATTTTCCTGA | 107 | 20 | 246 | 60 | 55 | 2 - 3 |
|  |  | Primer R | AGGTGGCAGGAATGTTCTGG |  | 20 | 431 | 59 | 55 |  |
| *Dbx2* | XM_001053826.4 | Primer F | ACTGAAGGAGTCACAGGTGAAG | 95 | 22 | 846 | 59 | 50 | 3 - 4 |
|  |  | Primer R | GGAGACACCTGTTGGAGAGC |  | 20 | 940 | 60 | 60 |  |
| *Disp3* | NM_001107992.2 | Primer F | AGCAGTCTACTAGCAAAGTCCA | 164 | 22 | 1391 | 58.83 | 45.45 | 3 - 4 / 4 - 5 |
|  |  | Primer R | GAAGGACAGGAACACTGAGCA |  | 21 | 1554 | 59.93 | 52.38 |  |
| *Gmr3* | NM_001105712.1 | Primer F | CAGTGTTTCCATACAGGTGGC | 180 | 21 | 676 | 59.19 | 52.38 | 5 - 6 |
|  |  | Primer R | GTCCAGTTGAAGAAGCGCAAG |  | 21 | 855 | 60.07 | 52.38 |  |
| *Igf1* | NM_001082477.2 | Primer F | CAGTGTTTCCATACAGGTGGC | 180 | 21 | 676 | 59.19 | 52.38 | 5 - 6 |
|  |  | Primer R | GTCCAGTTGAAGAAGCGCAAG |  | 21 | 855 | 60.07 | 52.38 |  |
| *Il1β* | NM_031512.2 | Primer F | CAGCTTTCGACAGTGAGGAGA | 139 | 20 | 91 | 59 | 45 | 2 - 3 |
|  |  | Primer R | TTGTCGAGATGCTGCTGTGA |  | 21 | 229 | 59 | 50 |  |
| *Rac2* | NM_001008384.1 | Primer F | TACACCACCAATGCCTTCCC | 172 | 20 | 164 | 59.96 | 55.00 | 3 - 4 |
|  |  | Primer R | TGAGGAACACGTCTGTCTGTG |  | 21 | 335 | 59.93 | 52.38 |  |
| *Rasgrp3* | NM_001108009.1 | Primer F | GAAGTTGGTTGAGCTCTGGG | 172 | 20 | 1576 | 58 | 55 | 12 - 13 / 14 - 15 |
|  |  | Primer R | AACACCTCATCCTGCACTGG |  | 20 | 1767 | 59 | 55 |  |
| *Siglec5* | NM_001106249.2 | Primer F | AAGGAGTCTCACAGGACAAATCA | 118 | 23 | 1348 | 59.35 | 43.48 | 6 - 7 / 7 - 8 |
|  |  | Primer R | TTCACTATGAAGAAGATGAGGCAG |  | 24 | 1465 | 58.33 | 41.67 |  |
| *Ppia* | NM_017101.1 | Primer F | AGGATTCATGTGCCAGGGTG | 118 | 20 | 216 | 60 | 50 | 6 - 7 / 7 - 8 |
|  |  | Primer R | CTCAGTCTTGGCAGTGCAGA |  | 20 | 402 | 59.9 | 45.45 |  |
