# Supplementary material for: Organotypic hippocampal culture model reveals differential responses to highly similar Zika virus isolates
Source: J Neuroinflammation. 2023 Jun 10;20:140. doi: 10.1186/s12974-023-02826-6 (PMC10257278; doi:10.1186/s12974-023-02826-6)
Supplement: Supplementary file 3 — Additional file 3. Relative quantification of ZIKV RNA by RT-qPCR. Viral load of OHC one and six days after PE243and SPH2015infection were compared pairwise with unpaired t test. [file 12974_2023_2826_MOESM3_ESM.docx]

**Additional File 3**

**Relative quantification of ZIKV RNA by RT-qPCR**

RNA extraction was performed using the QIAamp Viral RNA Mini Kit (Qiagen, Hilden, Germany). The primers utilized for the endogenous gene Peptidylprolyl Isomerase were as follows: Ppia F - CGC TAC GTT GCC TCT TAT CT and Ppia R - CAG CTC ACT GAT GAC CTT GT. For ZIKV detection, previously described primers targeting the virus envelope protein region were utilized (ZIKV 1086 and ZIKV 1162c) (Lanciotti et al., 2008). The reaction was carried out using the GoTaq Sybr 1-Step RT-qPCR System (Promega, Madison, WI). After total RNA extraction, reverse transcription was initiated with a 15-minute incubation at 37°C, followed by 10 minutes at 95°C. The PCR cycles consisted of the following conditions: 95°C for 10 seconds, 60°C for 30 seconds, and 72°C for 30 seconds, with a final step of a melting curve. The Master Mix utilized contained: 0.5 μL (500 nM) of Forward Primer, 0.5 μL (500 nM) of Reverse Primer, 0.4 μL of Reverse Transcriptase, 1.6 μL of Water, and 2.0 μL of each RNA sample. Relative quantification was obtained using 2^-∆∆Ct^ method.

Viral load of OHC one and six days after PE243 (n = 4) and SPH2015 (n = 3) infection were compared pairwise with unpaired t test. Data were expressed as mean ± standard deviation. * *P* ≤ 0.05.


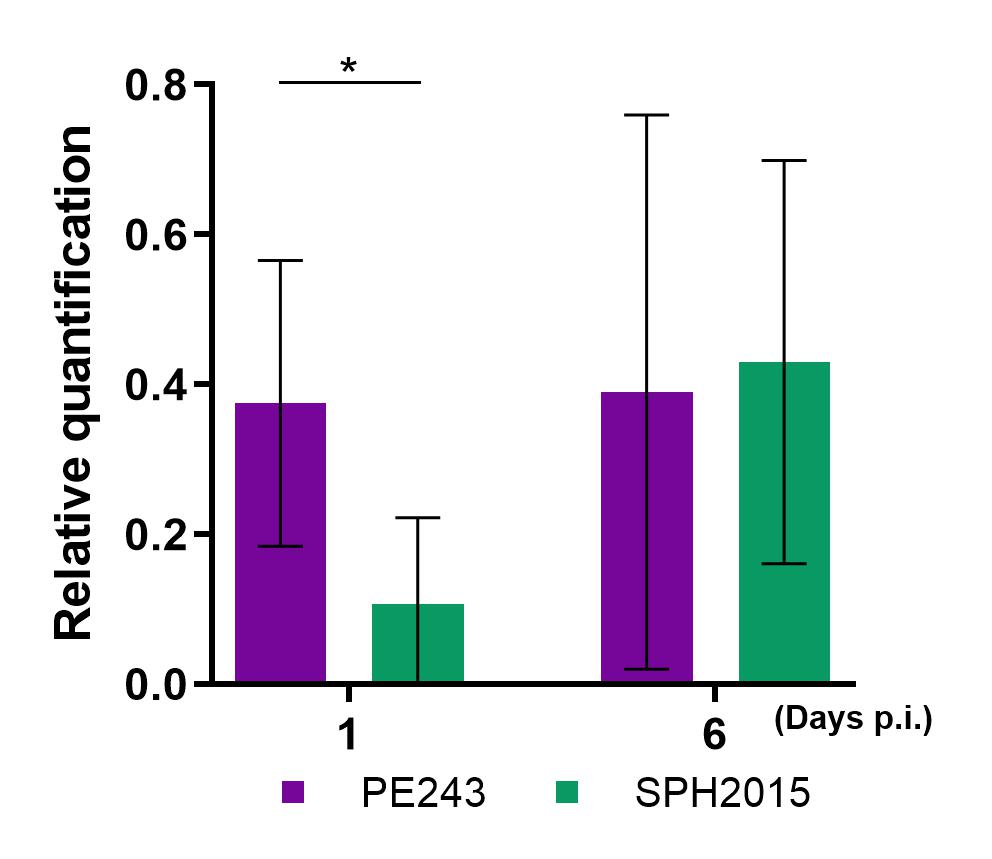


**Reference**:

Lanciotti, Robert S., et al. "Genetic and serologic properties of Zika virus associated with an epidemic, Yap State, Micronesia, 2007." *Emerging infectious diseases* 14.8 (2008): 1232.
