# Supplementary material for: Organotypic hippocampal culture model reveals differential responses to highly similar Zika virus isolates
Source: J Neuroinflammation. 2023 Jun 10;20:140. doi: 10.1186/s12974-023-02826-6 (PMC10257278; doi:10.1186/s12974-023-02826-6)
Supplement: Supplementary file 4 — Additional file 4. Dbx2 mRNA levels after 24 and 48 h of SPH2015 infection. Dbx2 mRNA levels) 24 and 48 h after SPH2015 infection were compared with two-tailed unpaired t test. Number of animals = 3 per group. Data distribution was evaluated using the Anderson–Darling, D’Agostino & Person, Shapiro–Wilk, or Kolmogorov–Smirnov tests. Data were expressed as mean ± standard deviation. ** P ≤ 0.01. [file 12974_2023_2826_MOESM4_ESM.docx]

**Additional File 4**

*Dbx2* mRNA levels (2^(-DCt)) 24 and 48 hours after SPH2015 infection were compared with two-tailed unpaired t test. Data distribution was evaluated using the Anderson-Darling, D'Agostino & Person, Shapiro-Wilk or Kolmogorov-Smirnov tests. Data were expressed as mean ± standard deviation. ** *P* ≤ 0.01.
