# Supplementary material for: Organotypic hippocampal culture model reveals differential responses to highly similar Zika virus isolates
Source: J Neuroinflammation. 2023 Jun 10;20:140. doi: 10.1186/s12974-023-02826-6 (PMC10257278; doi:10.1186/s12974-023-02826-6)
Supplement: Supplementary file 5 — Additional file 5. DEGs from OHC infected with ZIKV isolate PE243 16 h p.i. Genes expressed in response to the infection with PE243 or SPH2015 are highlighted in bold. [file 12974_2023_2826_MOESM5_ESM.docx]

**Additional File 5**

DEGs from OHCs infected with ZIKV isolate PE243 16h p.i. Genes expressed in response to the infection with PE243 or SPH2015 are highlighted in bold

| **PE243** | | | |
| --- | --- | --- | --- |
| Gene Symbol | Entrez Gene Name | Log2 Fold change | Expression *P* value |
| ***Adgre1*** | **adhesion G protein-coupled receptor E1** | **0,514** | **6,27E-05** |
| *Alpk3* | alpha kinase 3 | -0,415 | 2,17E-02 |
| *Ass1* | argininosuccinate synthase 1 | 0,498 | 2,32E-04 |
| *C1qa* | complement C1q A chain | -0,421 | 6,31E-03 |
| *C1qb* | complement C1q B chain | -0,423 | 5,89E-03 |
| *Caln1* | calneuron 1 | -0,402 | 2,14E-02 |
| *Cask* | calcium/calmodulin dependent serine protein kinase | -0,402 | 2,14E-02 |
| ***Ccl3l3*** | **C-C motif chemokine ligand 3 like 3** | **0,425** | **1,89E-02** |
| *Ccr5* | C-C motif chemokine receptor 5 | -0,565 | 6,27E-05 |
| *Cd34* | CD34 molecule | -0,428 | 2,77E-03 |
| *Cfh* | complement factor H | -0,410 | 2,39E-03 |
| *Clec7a* | C-type lectin domain containing 7A | -0,526 | 2,19E-04 |
| *Ctss* | cathepsin S | -0,488 | 1,59E-05 |
| ***Dbx2*** | **developing brain homeobox 2** | **-0,439** | **1,39E-02** |
| ***Disp3*** | **dispatched RND transporter family member 3** | **-0,507** | **2,19E-04** |
| *Drc7* | dynein regulatory complex subunit 7 | 0,409 | 4,35E-02 |
| *G0s2* | G0/G1 switch 2 | -0,401 | 3,11E-02 |
| *Gbp2* | guanylate binding protein 2 | 0,568 | 9,68E-05 |
| *Gpr34* | G protein-coupled receptor 34 | -0,443 | 1,44E-02 |
| ***Grm3*** | **glutamate metabotropic receptor 3** | **-0,416** | **3,15E-02** |
| *Grm5* | glutamate metabotropic receptor 5 | -0,437 | 6,86E-03 |
| ***Igf1*** | **insulin like growth factor 1** | **-0,970** | **4,56E-20** |
| ***Il1b*** | **interleukin 1 beta** | **0,423** | **2,14E-02** |
| *Lpl* | lipoprotein lipase | -0,498 | 2,16E-03 |
| *Mpeg1* | macrophage expressed 1 | -0,460 | 2,42E-04 |
| *Nrp1* | neuropilin 1 | -0,475 | 4,69E-03 |
| ***Rac2*** | **Rac family small GTPase 2** | **0,443** | **1,26E-02** |
| ***Rasgrp3*** | **RAS guanyl releasing protein 3** | **-0,428** | **1,39E-02** |
| ***Siglec8*** | **sialic acid binding Ig like lectin 8** | **0,422** | **2,95E-02** |
| *Slco3a1* | solute carrier organic anion transporter family member 3A1 | 0,432 | 2,36E-03 |
| *Sod2* | superoxide dismutase 2 | 0,517 | 6,27E-05 |
| *Trem2* | triggering receptor expressed on myeloid cells 2 | -0,535 | 3,62E-04 |
