# Supplementary material for: Organotypic hippocampal culture model reveals differential responses to highly similar Zika virus isolates
Source: J Neuroinflammation. 2023 Jun 10;20:140. doi: 10.1186/s12974-023-02826-6 (PMC10257278; doi:10.1186/s12974-023-02826-6)
Supplement: Supplementary file 6 — Additional file 6. DEGs from OHC infected with ZIKV isolate SPH2015 16 h p.i. Genes expressed in response to the infection with PE243 or SPH2015 are highlighted in bold. [file 12974_2023_2826_MOESM6_ESM.docx]

**Additional File 6**

DEGs from OHCs infected with ZIKV isolate SPH2015 16h p.i. Genes expressed in response to the infection with PE243 or SPH2015 are highlighted in bold

| **SPH2015** | | | |
| --- | --- | --- | --- |
| Gene Symbol | Entrez Gene Name | Log2 Fold change | Expression *P* value |
| ***Adgre1*** | **adhesion G protein-coupled receptor E1** | **0,634** | **3,37E-02** |
| *Adora1* | adenosine A1 receptor | -0,635 | 3,09E-02 |
| *Angpt1* | angiopoietin 1 | -0,643 | 1,26E-02 |
| *Ankrd66* | ankyrin repeat domain 66 | -0,647 | 1,21E-02 |
| *Aox1* | aldehyde oxidase 1 | -0,695 | 1,56E-02 |
| *Aplnr* | apelin receptor | -0,523 | 3,09E-02 |
| *Arhgap42* | Rho GTPase activating protein 42 | -0,342 | 3,14E-02 |
| *Bbox1* | gamma-butyrobetaine hydroxylase 1 | -0,667 | 2,78E-02 |
| *Ccdc153* | coiled-coil domain containing 153 | -0,598 | 3,60E-02 |
| *Ccdc190* | coiled-coil domain containing 190 | -0,636 | 3,33E-02 |
| *Ccdc88c* | coiled-coil domain containing 88C | -0,478 | 1,85E-02 |
| *Ccl2* | C-C motif chemokine ligand 2 | 1,337 | 1,45E-19 |
| ***Ccl3l3*** | **C-C motif chemokine ligand 3 like 3** | **0,680** | **1,21E-02** |
| *Ccl4* | C-C motif chemokine ligand 4 | 0,780 | 2,93E-03 |
| *Ccl7* | chemokine (C-C motif) ligand 7 | 0,631 | 3,58E-02 |
| *Cdh4* | cadherin 4 | -0,622 | 3,09E-02 |
| *Cdh6* | cadherin 6 | -0,607 | 1,98E-02 |
| *Cdk18* | cyclin dependent kinase 18 | -0,574 | 4,60E-02 |
| *Celsr1* | cadherin EGF LAG seven-pass G-type receptor 1 | -0,595 | 4,09E-02 |
| *Cfap57* | cilia and flagella associated protein 57 | -0,612 | 4,60E-02 |
| *Cfap91* | cilia and flagella associated protein 91 | -0,589 | 4,06E-02 |
| *Cfap99* | cilia and flagella associated protein 99 | -0,605 | 4,60E-02 |
| *Cldn14* | claudin 14 | -0,650 | 3,09E-02 |
| *Cspg5* | chondroitin sulfate proteoglycan 5 | -0,622 | 3,92E-02 |
| *Cxcl6* | C-X-C motif chemokine ligand 6 | 0,594 | 3,04E-02 |
| ***Dbx2*** | **developing brain homeobox 2** | **-0,710** | **1,30E-02** |
| *Diras2* | DIRAS family GTPase 2 | -0,724 | 7,16E-03 |
| ***Disp3*** | **dispatched RND transporter family member 3** | **-1,253** | **1,56E-09** |
| *Dkk3* | dickkopf WNT signaling pathway inhibitor 3 | -0,769 | 5,80E-04 |
| *Dnah11* | dynein axonemal heavy chain 11 | -0,689 | 1,80E-03 |
| *Dnah12* | dynein axonemal heavy chain 12 | -0,681 | 2,01E-02 |
| *Dnah9* | dynein axonemal heavy chain 9 | -0,741 | 3,28E-04 |
| *Dnai1* | dynein axonemal intermediate chain 1 | -0,616 | 3,58E-02 |
| *Dnajc18* | DnaJ heat shock protein family (Hsp40) member C18 | -0,394 | 2,49E-02 |
| *Draxin* | dorsal inhibitory axon guidance protein | -0,854 | 6,54E-04 |
| *Dusp15* | dual specificity phosphatase 15 | -0,629 | 3,15E-02 |
| *Ebi3* | Epstein-Barr virus induced 3 | 0,648 | 3,09E-02 |
| *Epb41l4b* | erythrocyte membrane protein band 4.1 like 4B | -0,671 | 7,47E-03 |
| *Ephx1* | epoxide hydrolase 1 | -0,656 | 2,72E-04 |
| *Erich3* | glutamate rich 3 | -0,608 | 4,80E-02 |
| *Fam107a* | family with sequence similarity 107 member A | -0,587 | 4,07E-02 |
| *Fam187a* | family with sequence similarity 187, member A | -0,648 | 3,04E-02 |
| *Fndc3c1* |  | -0,560 | 3,04E-02 |
| *Fzd1* | frizzled class receptor 1 | -0,706 | 8,28E-03 |
| *Gdpd2* | glycerophosphodiester phosphodiesterase domain containing 2 | -0,549 | 3,15E-02 |
| *Gpr37* | G protein-coupled receptor 37 | -0,599 | 3,05E-02 |
| ***Grm3*** | **glutamate metabotropic receptor 3** | **-0,700** | **2,93E-03** |
| *Hspa1a /hspa1b* | heat shock protein family A (Hsp70) member 1A | 0,531 | 3,82E-02 |
| *Hyal1* | hyaluronidase 1 | -0,432 | 3,04E-02 |
| ***Igf1*** | **insulin like growth factor 1** | **-0,708** | **2,93E-03** |
| ***Il1b*** | **interleukin 1 beta** | **0,661** | **1,91E-02** |
| *Itga5* | integrin subunit alpha 5 | 0,490 | 3,04E-02 |
| *Kcnj10* | potassium inwardly rectifying channel subfamily J member 10 | -0,684 | 1,91E-02 |
| *Kcnj16* | potassium inwardly rectifying channel subfamily J member 16 | -0,694 | 1,21E-02 |
| *Kcnn2* | potassium intermediate/small conductance calcium-activated channel, subfamily N, member 2 | -0,749 | 3,18E-03 |
| *Krt18* | keratin 18 | 0,624 | 3,09E-02 |
| *Lratd1* | LRAT domain containing 1 | -0,740 | 7,66E-03 |
| *Lrrc4* | leucine rich repeat containing 4 | -0,543 | 4,59E-02 |
| *Mgst1* | microsomal glutathione S-transferase 1 | -0,478 | 3,18E-03 |
| *Msi2* | musashi RNA binding protein 2 | -0,485 | 1,14E-02 |
| *Mybph* | myosin binding protein H | -0,623 | 3,82E-02 |
| *Myc* | MYC proto-oncogene, bHLH transcription factor | 0,445 | 2,87E-02 |
| *Nampt* | nicotinamide phosphoribosyltransferase | 0,381 | 3,18E-02 |
| *Nol4l* | nucleolar protein 4-like | -0,496 | 3,15E-02 |
| *Olfm3* | olfactomedin 3 | -0,778 | 2,91E-03 |
| *Olig1* | oligodendrocyte transcription factor 1 | -0,637 | 4,57E-03 |
| *P2rx6* | purinergic receptor P2X 6 | -0,612 | 4,01E-02 |
| *Pcdh10* | protocadherin 10 | -0,533 | 1,91E-02 |
| *Pcdh17* | protocadherin 17 | -0,458 | 4,19E-02 |
| *Pde9a* | phosphodiesterase 9A | -0,615 | 4,09E-02 |
| *Pdzrn3* | PDZ domain containing ring finger 3 | -0,575 | 3,60E-02 |
| *Pex5l* | peroxisomal biogenesis factor 5 like | -0,633 | 2,49E-02 |
| *Pitpnm3* | PITPNM family member 3 | -0,369 | 3,82E-02 |
| *Pkd1l1* | polycystin 1 like 1, transient receptor potential channel interacting | -0,526 | 4,60E-02 |
| *Plcd4* | phospholipase C delta 4 | -0,576 | 3,04E-02 |
| *Plcl1* | phospholipase C like 1 (inactive) | -0,542 | 4,60E-02 |
| *Plekha4* | pleckstrin homology domain containing A4 | 0,501 | 2,49E-02 |
| *Plxdc2* | plexin domain containing 2 | -0,704 | 1,21E-05 |
| *Plxna2* | plexin A2 | -0,434 | 2,93E-03 |
| *Pou3f2* | POU class 3 homeobox 2 | -0,602 | 2,08E-02 |
| ***Rac2*** | **Rac family small GTPase 2** | **0,607** | **4,60E-02** |
| *Raph1* | Ras association (RalGDS/AF-6) and pleckstrin homology domains 1 | 0,512 | 3,18E-02 |
| ***Rasgrp3*** | **RAS guanyl releasing protein 3** | **-0,724** | **3,65E-04** |
| *Sema3c* | semaphorin 3C | 0,540 | 3,37E-02 |
| *Serpine1* | serpin family E member 1 | 0,626 | 1,26E-02 |
| *Sez6* | seizure related 6 homolog | -0,697 | 1,21E-02 |
| *Sgcg* | sarcoglycan gamma | -0,566 | 4,08E-02 |
| *Siglec10* | sialic acid binding Ig like lectin 10 | 0,512 | 3,09E-02 |
| ***Siglec8*** | **sialic acid binding Ig like lectin 8** | **0,646** | **3,04E-02** |
| *Slc38a3* | solute carrier family 38 member 3 | -0,701 | 1,10E-03 |
| *Slc4a4* | solute carrier family 4 member 4 | -0,606 | 4,07E-02 |
| *Slc6a12* | solute carrier family 6 member 12 | 0,614 | 4,08E-02 |
| *Slco1c1* | solute carrier organic anion transporter family member 1C1 | -0,610 | 4,60E-02 |
| *Sord* | sorbitol dehydrogenase | -0,531 | 2,93E-03 |
| *Spag8* | sperm associated antigen 8 | -0,676 | 1,91E-02 |
| *Stpg1* | sperm tail PG-rich repeat containing 1 | -0,620 | 3,82E-02 |
| *Strn* | striatin | -0,405 | 3,18E-02 |
| *T2* | brachyury 2 | -0,650 | 3,04E-02 |
| *Tecta* | tectorin alpha | -0,591 | 4,07E-02 |
| *Tekt4* | tektin 4 | -0,634 | 3,33E-02 |
| *Thsd7a* | thrombospondin type 1 domain containing 7A | -0,494 | 4,08E-02 |
| *Tmeff2* | transmembrane protein with EGF-like and two follistatin-like domains 2 | -0,536 | 3,04E-02 |
| *Tmem98* | transmembrane protein 98 | -0,538 | 3,18E-03 |
| *Tspan5* | tetraspanin 5 | 0,454 | 1,64E-02 |
| *Tssc4* | tumor suppressing subtransferable candidate 4 | -0,392 | 3,04E-02 |
| *Ttll6* | tubulin tyrosine ligase like 6 | -0,638 | 3,04E-02 |
| *Tubb4a* | tubulin beta 4A class IVa | -0,664 | 1,36E-02 |
| *Vgll3* | vestigial like family member 3 | 0,602 | 4,19E-02 |
| *Wls* | Wnt ligand secretion mediator | -0,385 | 3,09E-02 |
| *Zbtb7c* | zinc finger and BTB domain containing 7C | -0,581 | 3,09E-02 |
| *Zfr2* | zinc finger RNA binding protein 2 | -0,611 | 2,97E-02 |
| *Zmynd12* | zinc finger MYND-type containing 12 | -0,607 | 4,84E-02 |
| *Znrf1* | zinc and ring finger 1 | 0,439 | 1,21E-02 |
