# Supplementary material for: Organotypic hippocampal culture model reveals differential responses to highly similar Zika virus isolates
Source: J Neuroinflammation. 2023 Jun 10;20:140. doi: 10.1186/s12974-023-02826-6 (PMC10257278; doi:10.1186/s12974-023-02826-6)
Supplement: Supplementary file 7 — Additional file 7. Real-time PCR validation of putative biomarkers identified with RNA-Seq. Gene expression values) at 16 h post infection for CTRL, PE243 and SPH2015 groups were compared with one-way Analysis of Variancetest followed by Tukey's multiple comparisons analysis. PE243 and SPH2015 were also compared separately with the CTRL group using Student's t testto identify possible statistically significant differences due to the statistical power of the multiple comparison method. Data distribution was evaluated using the Anderson–Darling, D’Agostino & Person, Shapiro–Wilk, or Kolmogorov–Smirnov tests. Outliers were identified by the ROUT test and removed from the analysis. Floating bars were expressed minimum and maximum values. Line at mean. Differences with P < 0.05 were considered statistically significant. * P ≤ 0.05; ** P ≤ 0.01; *** P ≤ 0.001; **** P ≤ 0.0001; Number of animals = CTRL; PE243; SPH2015. [file 12974_2023_2826_MOESM7_ESM.docx]

**Additional File 7**

Gene expression values (2^(-DCt)) at 16h post infection for CTRL, PE243 and SPH2015 groups were compared with one-way Analysis of Variance (ANOVA) test followed by Tukey's multiple comparisons analysis. PE243 and SPH2015 were also compared separately with the CTRL group using Student's t test (*P* value in gray) to identify possible statistically significant differences due to the statistical power of the multiple comparison method. Data distribution was evaluated using the Anderson-Darling, D'Agostino & Person, Shapiro-Wilk or Kolmogorov-Smirnov tests. Outliers were identified by the ROUT test and removed from the analysis. Floating bars were expressed minimum and maximum values. Line at mean. Differences with *P* < 0.05 were considered statistically significant. * *P* ≤ 0,05, ** *P* ≤ 0,01, *** *P* ≤ 0,001, **** *P* ≤ 0,0001. Number of animals = CTRL (4 to 6); PE243 (4 to 6); SPH2015 (4 to 6).
